# A secure multi-party computation protocol without CRS supporting multi-bit encryption

**DOI:** 10.1371/journal.pone.0265572

**Published:** 2022-03-18

**Authors:** Zong-Wu Zhu, Ru-Wei Huang

**Affiliations:** School of Computer and Electronic Information, Guangxi University, Nanning, China; University College of Engineering Tindivanam, INDIA

## Abstract

To solve the problems in the existing fully homomorphic encryption (FHE)-based secure multi-party computation (SMC) protocols such as low efficiency, the FHE scheme that supports multi-bit encryption was modified during the generation of the public key so that the users could generate their public keys independently without the common random string (CRS) matrix. Further, a multi-bit Gentry-Sahai-Waters scheme (MGSW) scheme without CRS was constructed. The modified LinkAlgo algorithm was adopted to expand the single-key ciphertext into the multi-key ciphertext and simplify the way of generating the expanded ciphertext. In this way, a multi-key FHE (MFHE) scheme was achieved based on the MGSW scheme. Finally, a three-round SMC protocol without CRS was constructed using the MFHE scheme and the decisional learning with errors (DLWE) assumption, which was secure in the semi-malicious model. Compared to the existing protocols, the protocol proposed herein can support multi-bit encryption and is found with smaller ciphertext size and lower storage overhead and generate the expanded ciphertext in a simpler way. Overall performance is better than existing protocols.

## 1. Introduction

Secure Multi-party Computation (SMC), a method proposed by Yao [1], can securely compute a function without disclosing its data. Each party can get its results but has no access to others’ data. SMC now consists of several constructors, including verifiable secret sharing [2], oblivious transfer [3], mix and match [4] and homomorphic encryption [5–7]. Devised by Rivest et al. [8] in 1978, Fully Homomorphic Encryption (FHE) supports direct encryption of the plaintext, and the same computation can be done on the ciphertext to get decryption results, namely, *f*(*Enc*(*m*)) = *Enc*(*f*(*m*)). Due to this unique feature, FHE has displayed great potential among all constructors of SMC protocols, and thus attracted greater attention over time. Since the launch of a FHE scheme by Gentry [9] in 2009, a wealth of similar schemes emerged, including DGHV10 [10], BV11 [11], BGV12 [12], Bra12 [13], GSW13 [14], BV14 [15], CM15 [5], and NK15 [16]. Meanwhile, scholars in China and abroad also substantially investigated FHE-based SMC protocols.

In 2012, López-Alt et al. [17] put forward the concept of multi-key fully homomorphic encryption (MFHE) for the first time, and made use of NTRU [18] to construct the first MFHE scheme. Therefore, an MFHE-based SMC protocol could be naturally created. Later, a lot of MFHE-based SMC protocols were constantly designed and improved. In 2016, Mukherjee et al. [6] established a two-round SMC protocol that achieved the best two-round interactions based on the GSW scheme, and proved that this scheme was secure in the malicious environment. The protocol needed to choose a common random string (CRS) matrix during the generation of a key, which undermined each user’s ability to generate their key independently. In the meantime, cascading and masking operations were added to the scheme, leading to a considerable volume of ciphertext matrix. Vijayakumar P et al. [19] proposed an efficient group key management technique that reduces the computational complexity without increasing the high storage complexity, thereby providing secure group communication in P2P networks. In the same year, a two-factor authentication scheme and a two-group key management scheme [20] were proposed to improve the security of vehicles communicating with a vehicle-mounted ad hoc network (VANET) environment. Audithan S et al. [21] proposed an anonymous authentication scheme to authenticate users, which is not easy to be maliciously accessed by attackers, and protects data transmitted in Internet business applications through mobile agents. In 2017, by increasing a new round of interaction and taking advantage of key homomorphism and threshold decryption, Wang Huiyong et al. [22] designed a simple three-round GSW-based SMC protocol with CRS, whose security was based on the Some-are-errorless LWE assumption, a variant of the LWE assumption. In this protocol, although a new round was added, the homomorphic computation depth and NAND gate complexity were reduced, and its overall efficiency was optimized compared to the MW16 scheme [6]. In 2018, Kim et al. [23] proposed the LinkAlgo algorithm to expand a single-key ciphertext into a multi-key one, and constructed for the first time a three-round SMC protocol without CRS that did not rely on a CRS matrix during the generation of a public key, allowing every user to generate their own public keys independently. This protocol met the multi-key CPA security requirements and could resist the attack of a semi-malicious adversary, but it was still slightly inferior to the protocol with CRS in terms of security and failed to prove that it was still secure in the malicious environment. In 2020, by employing the tool matrix and the encoding operations offered by Li Zengpeng [24] to improve the ciphertext expansion way of the KLP18 scheme [23], Tang Chunming et al. [25] built a three-round SMC protocol without CRS that outperformed the KLP18 scheme in efficiency, memory, and noise decryption, but it was only proven to be secure in the semi-malicious environment, as well. Dheerendra Mishra et al. [26] built a mutual authentication and key agreement scheme for mobile edge computing without the participation of trusted third parties, ensuring mutual authentication between users and edge servers and generating secure session keys. Vinoth R et al. [27] proposed a secure multi-factor authentication key agreement scheme for the Industrial Internet of Things (IIoT) to enable authorized users to remotely access sensing devices, effectively reducing communication during the authentication key agreement process and computational costs. In 2021, by referring to the multi-bit encryption scheme provided by Li Zengpeng, Tang Chunming et al. [28] pioneeringly developed a three-round SMC protocol with CRS that supports multi-bit encryption, whose security was based on the Ferr-LWE assumption and the Some-are-errorless LWE assumption, making it possible to resist the attack of an adversary in the malicious environment. In the same year, based on the multi-bit encryption scheme proposed by Li Zengpeng [24], Li Xixi et al. [29] modified his encryption algorithm and availed of the LinkAlgo algorithm to build a multi-bit multi-key FHE mechanism without CRS. Xia X et al. [30] proposed a cloud-assisted trustworthiness assessment mechanism and an efficient anonymous authentication and key agreement scheme based on non-interactive zero-knowledge to ensure privacy protection and data security of IoT devices in smart cities.

It can be seen from the above work that current FHE-based SMC protocols have been equipped with or without CRS. An SMC protocol with CRS is found with higher security. It is also proven to be secure by using the non-commutative zero-knowledge proof in the malicious environment. Still, it needs to choose a CRS matrix during the parameter setting phase. All users are required to generate their keys with the help of this matrix, which dramatically limits the users’ ability to generate keys independently. In contrast, an SMC protocol without CRS supports all users to generate their keys independently by eliminating the need for a trusted organization to distribute the CRS matrix. Nevertheless, the existing SMC protocols without CRS are still generally plagued by overlarge ciphertext, colossal memory space and low efficiency.

### 1.1 Objectives

To solve the problems of low efficiency of existing protocols, this paper converted the scheme of Chen Li et al. [31] into a multi-bit FHE scheme and constructed a multi-key homomorphic encryption scheme using the LinkAlgo algorithm. Finally, a three-round SMC protocol without CRS was designed to outperform all existing protocols in ciphertext size and storage overhead.

## 2. Knowledge base

### 2.1 Description of symbols

In this study, bold lowercase letters represent vectors, while bold capital letters refer to matrices. ℤ, ℝ, and ℤ_*q*_ refer to the set of integers, the set of real numbers, and the residue class ring of the integer modulo *q*. The length of the *n-*dimensional vector ***a*** is defined as its Euclidean norm $\|a\|=\sqrt{\sum_{i=0}^{n-1}a_{i}^{2}}$; the length of the vector set *S* is defined as ‖*S*‖ = max_*a*∈*S*_‖***a***‖. ***a***←*D* means that the variable ***a*** is randomly chosen from the Probability Distribution *D*; $a\overset{R}{\leftarrow }A$ means that the variable ***a*** is randomly and evenly chosen from the set A. The vector $a\in {\mathbb{Z}}_{q}^{n}$ can be expressed as ***a*** = (*a*_0_,⋯,*a*_*n*−1_); the polynomial *b*∈*R*_*q*_ can be written as *b* = (*b*_0_,⋯,*b*_*n*−1_). *c*_*i*_ refers to the *i*th row of the matrix ***C***; *I*_*n*_ represents the *n*-dimensional identity matrix; *φ*(*y*) means the probability *Pr*[*y*≤*x*|*y*~*N*(0,1)]. Unless otherwise specified, log*n* refers to log_2_*n*.

*O* and *o* suggest the computational complexity; also, for poly(⋅) and negl(⋅), if *f*(*n*) = *O*(*n*^*c*^), *f*(*n*) can be expressed as poly(n). If *f*(*n*) = *o*(*n*^−*c*^) holds for any constant c, *f*(*n*) can be expressed as negl(n), where n is a negligible function.

### 2.2 Definitions and theorems

**Definition 1 [32]** (LWE distribution): Define a secret vector $s\in {\mathbb{Z}}_{q}^{n}$, take uniform sampling $a\leftarrow {\mathbb{Z}}_{q}^{n}$, and choose *e*←*χ* wherein *χ* is a discrete Gaussian distribution on ℤ. The sample distribution *cA*_*s*,*χ*_ is outputted in the form of $(a,b=<a,s>+e(\mathrm{mod}q))\in {\mathbb{Z}}_{q}^{n}\times {\mathbb{Z}}_{q}$.

**Definition 2** (Searching LWE, abbreviated as SLWE): Define *m* independent samples $(a_{i},b_{i})\in {\mathbb{Z}}_{q}^{n}\times {\mathbb{Z}}_{q}$ from a given LWE distribution *A*_*s*,*χ*_ to output ***s***.

**Definition 3** (Decisional LWE, abbreviated as DLWE): For the security parameter *λ*, define *n* = *n*(*λ*) and *q* = *q*(*λ*)≥2, wherein *n* and *q* are integers. A distribution *χ* = *χ*(*λ*) on ℤ is defined. The *LWE*_*n*,*q*,*χ*_ problem is utilized to distinguish between the following two distributions: (1) Uniformly choose (*a*_*i*_,*b*_*i*_) from ${\mathbb{Z}}_{q}^{n+1}$; (2) Uniformly choose $s\leftarrow {\mathbb{Z}}_{q}^{n}$ and then take *e*_*i*_←*χ* for the uniform sampling $a_{i}\leftarrow {\mathbb{Z}}_{q}^{n}$. Supposing that *b*_*i*_ = <*a*_*i*_,*s*>+*e*_*i*_, $(a_{i},b_{i})\in {\mathbb{Z}}_{q}^{n}\times {\mathbb{Z}}_{q}$ is outputted. According to the *LWE*_*n*,*q*,*χ*_ assumption, the *LWE*_*n*,*q*,*χ*_ problem is hard to solve.

**Definition 4** The adversary models in the secure multi-party computation:

- Semi-honest model: All participants will strictly follow the protocol and not change the protocol or its data. However, the intermediate computation results may be maintained and used for computing the private data of other participants.
- Semi-malicious model: This model can be seen as an interactive Turing machine with reference and proof tapes. A semi-malicious adversary is obliged to record any data of a certain participant represented by it into the proof tape at any time. The adversary may decide whether to honestly execute the original protocol based on the inputs at random.
- Malicious model: All computational participants in this model can arbitrarily alter and disclose the protocol and its data, and even interfere with its normal performance.

**Definition 5 [31] *a*** and ***b*** are determined as the vectors along ${\mathbb{Z}}_{q}^{k}$; *k* is a positive integer; *q* is a modulo; *p* is the power of 2; *t* = ⌈log_*p*_*q*⌉; *N* =*kt*. The following function is defined:

$$\mathrm{mbDpt}(a)=a^{'}=(a_{1,1},\cdots ,a_{1,t},\cdots ,a_{k,1},\cdots ,a_{k,t})\in {\mathbb{Z}}_{p}^{N}$$ (1)

wherein ***a***’ is a *N*-dimensional vector $a_{i}=\sum_{j-1}^{t}a_{i,j}p^{j-1}$; *a*_*i*,*j*_∈ℤ_*p*_.


$$\mathrm{mbDp}t^{\left(-1\right)}(a^{'})=(\sum p^{j}\cdot a_{1,j},\cdots ,\sum p^{j}\cdot a_{k,j})$$ (2)

$$\mathrm{mbFlatten}(a^{'})=\mathrm{mbDpt}(mbDpt^{\left(-1\right)}(a^{'}))$$ (3)

$$pofmb(b)=(b_{1},pb_{1},\cdots ,p^{t-1}b_{1},\cdots ,b_{k},pb_{k},\cdots ,p^{t-1}b_{k})$$ (4)

**Theorem 1** Supposing that *e*_*i*_(*i*∈[*N*]) are a series of independent random variables subject to a certain bounded distribution *B*_*χ*_, the random variable $e=\frac{1}{N}\sum_{i=1}^{N}e_{i}$ is also subject to *B*_*χ*_.

**Theorem 2 [33]** For any *m*≥*n*⌈log*q*⌉, there is a matrix $G\in {\mathbb{Z}}_{q}^{n\times m}$ and its corresponding "short primary image" matrix function ***G***^−1^(⋅) to achieve ***G***^−1^(***M***)∈{0,1}^*m*×*m*’^ and ***GG***^−1^(***M***) = ***M*** for any matrix $M\in {\mathbb{Z}}_{q}^{n\times m^{'}}$, wherein *m*’ can be any number.

**Theorem 3 [31]** In terms of the MGSW scheme proposed herein, *L* is the maximum NAND gate depth of the circuit to be computed; in the absence of homomorphic computation, if ***C*** is the ciphertext obtained by encrypting 0, when $\left|\langle C_{m-1},s^{'}\rangle |<q/[4p{\left(pN+1\right)}^{L}\right]$, the scheme is correct.

**Proof.** By analyzing the correctness of homomorphic addition and multiplication, the noise is not greater than pN+1 times that of the original ciphertext after each homomorphic computation. As a result, when $\left|\langle C_{m-1},s^{'}\rangle |<q/[4p{\left(pN+1\right)}^{L}\right]$, $\left|\langle C_{m-1},s^{'}\rangle |<q/(4p\right)$ is achieved after no more than L homomorphic computations. According to the decryption algorithm, if $\left|\langle C_{m-1},s^{'}\rangle |<q/(4p\right)$, $\langle s^{'},C_{m-1}\rangle /(q/2p)<1/2$ is realized. Consequently, if the encrypted information is 0, 〈*C*_*m*−1_,***s***’〉 is closer to 0 than *q*/(2*p*), and $\mu =\lfloor \langle s^{'},C_{m-1}\rangle /(q/2p)+1/2\rfloor \mathrm{mod}2<\lfloor 1/2+1/2\rfloor \mathrm{mod}2=0$; when the opposite happens, the correctness of the scheme can be guaranteed.

## 3. MGSW scheme that supports multi-bit encryption

The MGSW scheme constructed in this section is a modified FHE scheme that supports multi-bit encryption based on Literature [31], whose security is based on the DLWE assumption. Each user in Literature [31] relies on the CRS matrix ***A*** to generate their public keys during the key generation process, undermining their ability to generate a public key independently. In contrast, our scheme is more advanced because the participants do not need any CRS matrix to generate public keys; instead, each of them can generate his/her public keys independently by randomly choosing a matrix ***A*** from ${\mathbb{Z}}_{q}^{n\times n}$. The scheme is detailed as follows:

For the given modulo *q* and the dimension *N*, the ciphertext ***C*** is an *N*×*N* dimension matrix defined on ℤ_*p*_, and each matrix component is far less than *q*. The secret key ***sk*** of ***C*** is defined as an *N*-dimension vector along ℤ_*p*_. Supposing that the plaintext *μ* is a small integer, when ***C***⋅***sk*** = *μ*⋅***sk***+***e***, ***C*** is defined as the ciphertext of *μ* wherein ***e*** is a small error vector. During the decryption process, first take the *i*th row ***C***_*i*_ of ***C***, compute *x*←〈***C***_*i*_,***sk***〉 = *μ*⋅*sk*_*i*_+*e*_*i*_, and output *μ* = ⌊*x*/*sk*_*i*_⌉, wherein *sk*_*i*_ is the *i*th element of ***sk***, *e*_*i*_ is the *i*th element of ***e***, and *i*∈[0,*N*−1]. The information *μ* can be considered an eigenvalue of the ciphertext matrix ***C***, while the secret key ***sk*** is the approximate eigenvector of ***C*** corresponding to the eigenvalue *μ*.

MGSW.Setup(1^*λ*^,1^*L*^): *λ* is the security parameter; *L* is the maximum NAND gate depth of the circuit; the lattice dimension *n* = *n*(*λ*,*L*); for the modulo *q*, the error distribution *χ* = *χ*(*λ*,*L*); *m* = *m*(*λ*,*L*) = *O*(*n*log*q*). By choosing appropriate parameters, the LWE assumption holds, thus outputting params = (*n*,*q*,*χ*,*m*).

MGSW.Keygen(n,q): For the positive integer n, take the depth of homomorphic computation as l, randomly and uniformly choose $A\overset{R}{\leftarrow }{\mathbb{Z}}_{q}^{n\times n}$ from ${\mathbb{Z}}_{q}^{n\times n}$, and take the sample **s** from the discrete Gaussian distribution χ^n×l^ on ℤ^n×l^. The public key $pk=(A,b=A\cdot s+e)\in {\mathbb{Z}}_{q}^{n\times n}\times {\mathbb{Z}}_{q}^{n}$ is computed, and the secret key is $sk=(\begin{array}{c} -s\\ 1 \end{array})\in {\mathbb{Z}}_{q}^{n+1}$.

MGSW.Encrypt(*pk*,*μ*): For the plaintext *μ*∈ℤ_*p*_, randomly and uniformly choose ***r***_*i*_, *e*_*i*,1_←*χ*^*n*^, *e*_*i*,2_←*χ* and *i* = 1,⋯,(*n*+1)⋅*t*, compute $C_{i,1}=A^{T}\cdot r_{i}+e_{i,1}\in {\mathbb{Z}}_{q}^{n}$ and *C*_*i*,2_ = ***b***^*T*^⋅***r***_2_+*e*_*i*,2_∈ℤ_*q*_. Supposing that *c*’ is a matrix composed of *m* = (*n*+1)⋅*t* ciphertexts arranged as column vectors, whose dimension is (*n*+1)×*m*, thus outputting the ciphertext $C=mbFlatten(\mu \cdot I_{N}+mbDpt(C^{'}))\in {\mathbb{Z}}_{p}^{m\times m}$.

MGSW.Decrypt(***sk***,***C***): For the ciphertext $C\in {\mathbb{Z}}_{p}^{m\times m}$ and the secret key $sk=(\begin{array}{c} -s\\ 1 \end{array})\in {\mathbb{Z}}_{q}^{n+1}$, supposing that *s*’ = pofmb(***sk***), compute and output the plaintext $\mu =\lfloor \langle s^{'},C_{m-1}\rangle /(q/2p)+\frac{1}{2}\rfloor \mathrm{mod}p$.

MGSW.Add(*C*_1_,*C*_2_): Input the ciphertexts *C*_1_ and *C*_2_, and output the new ciphertext ***C*** = mbFlatten(*C*_1_+*C*_2_) after the homomorphic addition.

MGSW.Mult(*C*_1_,*C*_2_): Input the ciphertexts *C*_1_ and *C*_2_, and output the new ciphertext ***C*** = mbFlatten(*C*_1_⋅*C*_2_) after the homomorphic multiplication.

### 3.1 Correctness

During multi-bit encryption and decryption, in the absence of homomorphic computation, if the plaintext is *μ*’, we can obtain $\mu =\lfloor \langle s^{'},C_{m-1}\rangle /(q/2p)+\frac{1}{2}\rfloor \mathrm{mod}p=\lfloor \mu^{'}+e/(q/2p)+\frac{1}{2}\rfloor \mathrm{mod}p$ after the decryption according to the encryption and decryption processes; when |*e*/(*q*/2*p*)<1/2|, *μ* = *μ*’, so it is correctly decrypted. For homomorphic addition, ***C*** = mbFlatten((*μ*_1_+*μ*_2_)⋅***I***_*N*_+mbDmp(*C*_1_+*C*_2_)), and MGSW.Dec(***sk*,*C***) = (*μ*_1_+*μ*_2_)mod*p*. For homomorphic multiplication, ***C***⋅***sk*** = *μ*_1_⋅*μ*_2_⋅***sk***+*μ*_2_⋅***e***_1_+*C*_1_⋅***e***_2_, and MGSW.Dec(***sk*,*C***) = *μ*_1_⋅*μ*_2_. Therefore, correct decryption is achieved.

After homomorphic multiplication, since the coefficient of both *μ*_2_ and *C*_1_ are limited to ℤ_*p*_, the noise is not greater than *pN*+*p* times that of the original ciphertext. Consequently, during the multi-bit encryption, the limitation exerted by Theorem 3 on the noise becomes $\left|\langle C_{m-1},s^{'}\rangle |<q/[4p{\left(pN+p\right)}^{L}\right]$. Considering *pN* = *pkt*≫*p*, the influence of this change on the modulo *q* can be ignored.

### 3.2 Security

**Theorem 4** Supposing that the parameters *n* = poly(*λ*) and *q* = poly(*λ*) constitute a polynomial with the security parameter *λ*, and that the Attacker can distinguish the ciphertext of the MGSW scheme and the uniform distribution on ${\mathbb{Z}}_{p}^{m\times m}$ with a non-ignorable advantage, the DLWE problem is also solved. Therefore, if it is supposed that this problem is hard to solve, the MGSW scheme can meet the plaintext’s (IND-CPA) security criteria.

**Proof.** The Theorem is proven by defining the following game sequences:

a) Game0.

Initialization: The Challenger runs MGSW.Keygen(*n*,*q*) to generate the public-secret key pair (pk, sk), and gives the public key to the Attacker $\tilde{A}$.

Step 1: The Attacker may encrypt the information *μ*∈{0,1} independently or through the Challenger. If the latter method is adopted, the Challenger needs to return the ciphertext accurately.

Challenge: At a given time, the Attacker challenges the Challenger and sends the challenge plaintext *μ*_1_,*μ*_2_∈{0,1}. By randomly choosing *b*∈{0,1}, the Challenger runs MGSW.Encrypt(*pk*,*μ*), computes and sends the challenge ciphertext ***C*** to the Challenger.

Step 2: Just as done in Step 1, the Attacker may encrypt the information *μ*∈{0,1} independently or through the Challenger.

Guessing: The Attacker guesses the challenge plaintext chosen from the challenge step, and outputs *b*’∈{0.1}. Thus, Game0 is a classic IND-CPA attacking game where the Attacker’s advantage is marked as:

$$Adv_{CPA}(\tilde{A})=|\mathrm{Pr}[\tilde{A}(pk,MGSW.Encrypt(pk,\mu_{0}))=1]-\mathrm{Pr}[\tilde{A}(pk,MGSW.Encrypt(pk,\mu_{1}))=1]|$$ (5)

b) Game1.

In Game1, except for the initialization period, the Challenger follows all other steps as with Game0.

Initialization: The Challenger randomly and uniformly chooses the public key $A\overset{R}{\leftarrow }{\mathbb{Z}}_{q}^{(n+1)\times n}$ and gives it to the Attacker $\tilde{A}$.

The Attacker’s advantage in Game1 is marked as $Adv_{Game1}(\tilde{A})$. In Game1, the public key is no longer generated through the secret key. As the public key in Game0 can be seen as *n*×1 *LWE*_*q*,*n*,*χ*_ instances, the public key in Game1 is randomly chosen from the uniform distribution. Therefore, if the *DLWE*_*q*,*n*,*n*,*χ*_ problem can be solved via the non-ignorable advantage, Game0 and Game 1 can also be distinguished based on the same edge. If the assumption *DLWE*_*q*,*n*,*n*,*χ*_ holds, the advantage of the Attacker to distinguish between Game0 and Game1 is negligible, thus getting

$$Adv_{CPA}(\tilde{A})=Adv_{Game1}(\tilde{A})+negl(\lambda )$$ (6)

c) In Game2, except for the challenge period, the Challenger follows all other steps as with Game1. At a given time, the Attacker challenges the Challenger and sends the challenge plaintext *μ*_1_,*μ*_2_∈{0,1}. The Challenger randomly chooses *b*∈{0,1} and $C\overset{R}{\leftarrow }{\mathbb{Z}}_{q}^{n+1}$, and sends the challenge ciphertext *c*’ =*c*+(*o*,*μ*⋅⌊*q*/2⌋) to the Attacker. The advantage of the Attacker in Game2 is marked as $Adv_{Game2}(\tilde{A})$. Likewise, $Adv_{Game1}(\tilde{A})\leq Adv_{Game2}(\tilde{A})+negl(\lambda )$.

d) In Game3, except for the challenge period, the Challenger follows all other steps as with Game2. The Challenger sends the challenge ciphertext to the Attacker. The advantage of the Attacker in Game3 is marked as $Adv_{Game3}(\tilde{A})$. In Game3, both the public key and the challenge ciphertext are taken from uniform distributions, and do not contain any information of the plaintext, so $Adv_{Game3}(\tilde{A})=0$. Since C in both Game2 and Game3 is taken from the uniform distribution on ${\mathbb{Z}}_{q}^{n+1}$, ***C’*** in Game2 and ***C*** in Game3 are statistically indistinguishable, namely, $Adv_{Game2}(\tilde{A})\leq Adv_{Game3}(\tilde{A})+negl(\lambda )$.

As $Adv_{Game3}(\tilde{A})=0$, if the assumption *DLWE*_*q*,*n*,2*n+*1,*χ*_ holds, $Adv_{CPA}(\tilde{A})=negl(\lambda )$, and the MGSW scheme meets the IND-CPA security criteria.

## 4. Modified LinkAlgo algorithm

It is often the case that the construction of a multi-key FHE scheme relies on the homomorphic computation of the ciphertexts under different keys, but the MGSW scheme proposed herein can just generate the multi-bit single-key ciphertext. Consequently, in our scheme, the LinkAlgo algorithm [23] is adopted for expanding the multi-bit single-key ciphertext into the multi-bit multi-key ciphertext. As mentioned in the KLP18 scheme [23], the expansion of multi-key ciphertext involves complicated steps, leading to low efficiency, high memory space and loud decryption noise. Therefore, the complicated ciphertext expansion way is optimized herein to get simpler expanded ciphertext, further increasing the efficiency. The modified LinkAlgo algorithm is detailed as follows:

For the matrix **R**∈{0,1}^*m*×*m*^, **V**^(*s*,*t*)^ is the *β* noise ciphertext of **R**^(*s*,*t*)^ encrypted with the GSW encryption algorithm under $\left(pk,sk)=(K,\bar{t}\right)$. Supposing that $\left(pk^{'},sk^{'})=(K^{'},{\bar{t}}^{'}\right)$ is another pair of keys, by inputting *pk*’ and all **R**^(*s*,*t*)^ into the modified LinkAlgo algorithm, **Y** is outputted to achieve **tY** = **tK**’**R**+***e*** and ‖***e***‖_∞_≤*m*^3^*β* (***e*** is the noise), thus outputting the optimized expanded ciphertext ${\hat{C}}_{i}$.

Modified LinkAlgo Algorithm

Input: *pk*’ and {**R**^(*s*,*t*)^}_*s*,*t*∈[*m*]_

Output: ${\left\{V_{i,j}{}^{\left(s,t\right)}\right\}}_{s,t\in [m]}\leftarrow \{GSW.Enc(R^{\left(s,t\right)},pk_{j})\}s,t\in [m],j\in [t]$

Output: $Y_{i}^{j}\in {\mathbb{Z}}_{q}^{n\times m},j\in [t]$

1. Define $L_{s,t}\in {\mathbb{Z}}_{q}^{n\times m}$, wherein *s*,*t*∈[*m*]

$$L_{s,t}[a,b]=\{\begin{array}{c} K^{'}[a,s];t=b\\ 0;other \end{array}$$

2. Output $Y=\sum_{s=1}^{m}\sum_{t=1}^{m}V^{\left(s,t\right)}G^{-1}(L_{s,t})\in {\mathbb{Z}}_{q}^{n\times m}$

Output:

$${\hat{C}}_{i}=[\begin{array}{ccccc} C_{i}-Y_{i}^{1} & 0 & \cdots & 0 & 0\\ 0 & C_{i}-Y_{i}^{2} & \cdots & 0 & 0\\ \vdots & \vdots & \vdots & \vdots & \vdots \\ Y_{i}^{i} & \cdots & C_{i} & \cdots & Y_{i}^{i}\\ \vdots & \vdots & \vdots & \vdots & \vdots \\ 0 & 0 & \cdots & 0 & C_{i}-Y_{i}^{t} \end{array}]$$

Now, by defining **tY** = **tK**’**R**+***e***, the following detailed processes are presented to prove that ‖***e***‖_∞_≤*m*^3^*β* holds:

$$tY=\sum_{s,t}tV^{\left(s,t\right)}G^{-1}(L_{s,t})$$

$$=\sum_{s,t}\left(tR^{\left(s,t\right)}G+e_{s,t}\right)G^{-1}(L_{s,t})$$

$$=\sum_{s,t}\left(tR^{\left(s,t\right)}L_{s,t}+e_{s,t}^{'}\right)$$

$$=t\sum_{s,t}R^{\left(s,t\right)}L_{s,t}+\sum_{s,t}^{m}e_{s,t}^{'}$$ (7)

wherein $e_{s,t}^{'}=e_{s,t}G^{-1}(L_{s,t})$ and $\|e_{s,t}^{'}\|\leq m\beta$.

Now, it is time to prove $\sum_{s=1}^{m}\sum_{t=1}^{m}R^{\left(s,t\right)}L_{s,t}=K^{'}R$

$$\sum_{s=1}^{m}\sum_{t=1}^{m}R^{\left(s,t\right)}L_{s,t}=\sum_{s=1}^{m}\sum_{t=1}^{m}\left(\begin{array}{ccccc} 0 & \cdots & R^{\left(s,t\right)}K^{'(1,s)} & \cdots & 0\\ \vdots & \cdots & R^{\left(s,t\right)}K^{'(2,s)} & \cdots & 0\\ \vdots & \vdots & \vdots & \cdots & \vdots \\ 0 & \cdots & R^{\left(s,t\right)}K^{'(n,s)} & \cdots & 0 \end{array}\right)$$

$$=\sum_{t=1}^{m}\left(\begin{array}{ccccc} 0 & \cdots & \sum_{s=1}^{m}R^{\left(s,t\right)}K^{'(1,s)} & \cdots & 0\\ \vdots & \cdots & \sum_{s=1}^{m}R^{\left(s,t\right)}K^{'(2,s)} & \cdots & 0\\ \vdots & \vdots & \vdots & \cdots & \vdots \\ 0 & \cdots & \sum_{s=1}^{m}R^{\left(s,t\right)}K^{'(n,s)} & \cdots & 0 \end{array}\right)$$

$$=\sum_{t=1}^{m}\left(\begin{array}{ccccc} 0 & \cdots & \langle K_{1}^{'row},R_{t}^{col}\rangle & \cdots & 0\\ \vdots & \cdots & \langle K_{2}^{'row},R_{t}^{col}\rangle & \cdots & 0\\ \vdots & \vdots & \vdots & \cdots & \vdots \\ 0 & \cdots & \langle K_{m}^{'row},R_{t}^{col}\rangle & \cdots & 0 \end{array}\right)=K^{'}R$$ (8)

Therefore, **tY** = **tK**’**R**+***e***, wherein $e=\sum_{s=1}^{m}\sum_{t=1}^{m}e_{s,t}^{'}$ and ‖***e***‖_∞_≤*m*^3^*β*.

## 5. Multi-key Fully Homomorphic Encryption (MFHE)

In this section, based on the above MGSW scheme that supports multi-bit encryption, the modified LinkAlgo algorithm is adopted to expand the multi-bit single-key ciphertext to the multi-bit multi-key ciphertext and construct an MFHE scheme. To be specific, G and G^−1^(⋅) are the same as those described in Theorem 2; G is expanded into ${\hat{G}}_{t}=diag(G,\cdots G)\in {\mathbb{Z}}_{q}^{nN\times mN}$, and ${\hat{G}}_{t}^{-1}(\cdot )$ is its corresponding function. This scheme is composed by the polynomial algorithm MFHE = (Sepup, Keygen, Enc, Expand, Eval, Dec) for a series of probability events, as detailed below:

−MFHE.Setup(1^*λ*^,1^*L*^)→(params)

1. Run MGSW.Setup(1^*λ*^,1^*L*^)
2. Output params = (*n*,*q*,*χ*,*m*)

−MFHE.Keygen(params)→(*pk*,*sk*)

1. Run MGSW.Keygen(*n*,*q*)
2. Output $\left(pk,sk)=(K,\bar{t}\right)$

−MFHE.Enc(*pk*,*μ*)→***C***

1. Run MGSW.Encrypt(*pk*,*μ*)
2. Output ***C*** = mbFlatten(*μ*⋅***I***_*N*_+mbDpt(***C***’))

$-MFHE.Expand((pk_{1},pk_{2},\cdots ,pk_{t}),i,C)\to ({\hat{C}}_{i})$

After the modified LinkAlgo algorithm is adopted to input the key and new ciphertext, the expanded ciphertext is outputted as:

$${\hat{C}}_{i}=[\begin{array}{ccccc} C_{i}-Y_{i}^{1} & 0 & \cdots & 0 & 0\\ 0 & C_{i}-Y_{i}^{2} & \cdots & 0 & 0\\ \vdots & \vdots & \vdots & \vdots & \vdots \\ Y_{i}^{i} & \cdots & C_{i} & \cdots & Y_{i}^{i}\\ \vdots & \vdots & \vdots & \vdots & \vdots \\ 0 & 0 & \cdots & 0 & C_{i}-Y_{i}^{t} \end{array}]$$

$-MFHE.Eval(params,f,{\hat{C}}_{1},\cdots ,{\hat{C}}_{l})\to ({\hat{C}}^{*})$

1. Present *l* expanded ciphertexts ${\hat{C}}_{i}(i=1,\cdots ,l)$ and the matrix functions ${\hat{G}}_{t}^{-1}(\cdot )$ corresponding to ${\hat{G}}_{t}=diag(\hat{G},\cdots ,\hat{G})$
2. Output ${\hat{C}}^{*}$

$-MFHE.Dec(params,({\bar{t}}_{1}\cdots {\bar{t}}_{N},),{\hat{C}}_{i})\to (\mu )$

1. Present the key $\bar{t}=({\bar{t}}_{1},\cdots ,{\bar{t}}_{N})$ and an expanded ciphertext ${\hat{C}}_{i}$, and define $\hat{w}=[\underset{mt↑}{\underset{︸}{0,\cdots ,0}},\underset{t↑}{\underset{︸}{\left\lceil q/2\rceil ,\cdots ,\lceil q/2\right\rceil }}]\in {\mathbb{Z}}_{q}^{(m+1)t}$
2. Run MGSW.Decrypt(***sk*,*C***)
3. Output *μ*

Threshold decryption can be implemented on the above-expanded ciphertext ${\hat{C}}_{i}$, as detailed below:

$‐MFHE.PartDec(C,{\bar{t}}_{i})\to (p_{i})$

1. Present an expanded ciphertext $\hat{C}$ and the *i*th key ${\bar{t}}_{i}\in {\mathbb{Z}}_{q}^{(m+1)\times t}$, and divide the ciphertext $\hat{C}$ into *N*-row submatrices ${\hat{C}}_{i}(\hat{C}=({\hat{C}}_{1}^{T},\cdots ,{\hat{C}}_{N}^{T}))$ wherein ${\hat{C}}_{i}^{T}\in {\mathbb{Z}}_{q}^{(m+1)\times tN}$. Define $\hat{w}=[\underset{mt↑}{\underset{︸}{0,\cdots ,0}},\underset{t↑}{\underset{︸}{\left\lceil q/2\rceil ,\cdots ,\lceil q/2\right\rceil }}]\in {\mathbb{Z}}_{q}^{(m+1)t}$

2. Compute $\gamma_{i}={\bar{t}}_{i}{\hat{C}}_{i}{\hat{G}}_{t}^{-1}({\hat{w}}^{T})$

3. Output ***p***_*i*_ = ***γ***_*i*_+***e***_*i*_, wherein ***e***_*i*_ is a small noise.

MFHE.FinDec(***p***_1_,⋯,***p***_*t*_)→(*μ*)

1. Input ***p***_1_,⋯,***p***_*t*_, and compute $p=\sum_{i=1}^{t}p_{i}$
2. Output $\mu =|\left\lfloor \frac{p}{q/2}\right\rfloor |$.

### 5.1 Correctness of expanded ciphertext

${\hat{C}}_{i}\leftarrow MFHE.Expand((pk_{1},pk_{2},\cdots ,pk_{N}),i,C)$ can be obtained by expanding the ciphertext of the *i*th user, wherein ***C*** is the multi-key ciphertext of the plaintext *μ* after being encrypted by the MGSW scheme under $\left(K,{\hat{t}}_{i}\right)$. By defining the multi-key $\hat{t}=({\hat{t}}_{1},\cdots ,{\hat{t}}_{N})$ and the public matrix $\hat{G}$, if $\hat{C}$ satisfies $\hat{t}{\hat{C}}_{i}=\mu \hat{t}{\hat{G}}_{N}$ wherein $M_{t}=diag(\underset{t↑}{\underset{︸}{M,\cdots ,M}})$, it is natural for us to promote the MGSW scheme. Next, the correctness of the expanded ciphertext will be proven:

$$\hat{t}{\hat{C}}_{i}=[t_{1}(C‐Y_{i}^{1})+t_{i}Y_{i}^{i},t_{2}(C‐Y_{i}^{2})+t_{i}Y_{i}^{i},\cdots ,t_{i}Y,\cdots ,t_{N}(C‐Y_{i}^{N})+t_{i}Y_{i}^{i}]$$

$$=[t_{1}C‐t_{1}Y_{i}^{1}+t_{i}Y_{i}^{i},t_{2}C‐t_{2}Y_{i}^{2}+t_{i}Y_{i}^{i},\cdots ,t_{i}C,\cdots ,t_{N}C‐t_{N}Y_{i}^{N}+t_{i}Y_{i}^{i}]$$

For all *j*≠*i*, $t_{j}C‐t_{j}Y_{i}^{j}+t_{i}Y_{i}^{i}=t_{j}K_{i}M_{i}+\mu t_{i}G‐t_{j}K_{i}M_{i}‐e_{j}+t_{i}K_{i}M+e_{i}=\mu t_{j}G+{\tilde{e}}_{j}$

Therefore, $\hat{t}{\hat{C}}_{i}=\mu \hat{t}{\hat{G}}_{N}+e$, $e=[{\tilde{e}}_{1},\cdots ,{\tilde{e}}_{2},\cdots ,{\tilde{e}}_{N}]$.

To be specific, ${\bar{e}}_{j}=-e_{j}+e_{i}+t_{i}K_{i}M_{i}\leq B_{\chi }+mB_{\chi }+B_{\chi }=(m+2)B_{\chi }$. $e_{i}^{'}=t_{i}C-\mu t_{i}G$, and ${\left\|e_{i}^{'}\right\|}_{\infty }\leq mB_{\chi }$.

Therefore, ‖*e*‖_∞_≤(*m*+2)*B*_*χ*_. To get it correctly decrypted, the condition (*m*+2)*B*_*χ*≤_*q*/(4*mN*) shall be met. An appropriate parameter q can be chosen to achieve the result that (*m*+2)*B*_*χ*≤_*q*/(4*mN*) holds.

### 5.2 Security of the proposed scheme

a) First of all, the scheme encryption resembles that of the above MGSW scheme constructed. It can be known from the security of the MGSW scheme that this encryption process meets the IND-CPA security criteria.

b) Security of the expanded ciphertext. The expanded ciphertext is obtained from the LinkAlgo algorithm where ***Y*** is generated by ***V***^(*s*,*t*)^ and ***G***^−1^(***L***_*s*,*t*_), as ***G***^−1^(***L***_*s*,*t*_) means the bit decomposition of ***L***_*s*,*t*_ and ***V***^(*s*,*t*)^ refers to the encryption of ***R***^(*s*,*t*)^. It can be seen from Literature [13] that ***V***^(*s*,*t*)^***G***^−1^(***L***_*s*,*t*_) and the matrix uniformly chosen from ${\mathbb{Z}}_{q}^{(m+1)\times N}$ are indistinguishable in terms of computation, so this expanded ciphertext is secure.

Therefore, the proposed scheme herein is proven to be secure.

## 6. Three-round Secure Multi-party Computation (SMC) protocol

This section utilizes the above MFHE scheme to construct a three-round SMC protocol without CRS that supports multi-bit encryption. Although the best two-round interactions have been realized in the protocol with CRS in Literature [6], an SMC protocol without CRS requires at least three rounds to complete the entire protocol: As no CRS matrix is used in the protocol, each participant generates his/her key pair independently, and distributes the key before the protocol comes into force, which takes at least one round; it also takes another two rounds to generate and release ciphertext and compute and release the partially decrypted result. As a result, an SMC protocol with CRS requires at least three rounds.

*π*_*f*_: In the SMC protocol without CRS, the single-valued function *f* is securely solved. The protocol is secure in the semi-honest and semi-malicious models, as detailed below:

**Preprocessing:** Set up the parameters (params)←MFHE.Setup(1^*λ*^,1^*L*^) to make sure that all participants share the parameter settings.

**Input:** For *i*∈[*N*], each participant *P*_*i*_ inputs the private data *x*_*i*_∈{0,1} to compute the function *f*({0,1}^*N*^→{0,1}), wherein *L* is the circuit depth of *f*.

**Round 1**. For each participant *P*_*i*_, the following steps are carried out.

- Generate (*pk*_*i*_,*sk*_*i*_)←MFHE.Keygen(params).

- Release the public key {*pk*_*i*_}_*i*∈[*N*]_.

**Round 2**. Each participant *P*_*i*_ receives the public key {*pk*_*i*_}_*i*∈[*N*]\{*i*}_ from others and follows the steps below.

- Use the public key *pk*_*i*_ to encrypt the plaintext *m’* and get the ciphertext ***C***←MFHE.Enc(*pk*,***m***’).

- Run the expanded algorithm to get the expanded ciphertext ${\hat{C}}_{i}\leftarrow MFHE.Expand((pk_{1},pk_{2},\cdots ,pk_{t}),i,C)$, and then release the expanded ciphertext ${\hat{C}}_{i}$.

**Round 3**. Each participant *P*_*i*_ receives the ciphertext ${\hat{C}}_{i}$ from others and follows the steps below.

- Output the assessed ciphertext ${\hat{C}}^{*}\leftarrow MFHE.Eval(params,f,{\hat{C}}_{1},\cdots ,{\hat{C}}_{l})$ after homomorphic computation.

- Threshold encryption is implemented. Get the partially decrypted result $p_{i}\leftarrow MFHE.PartDec(C,{\bar{t}}_{i})$, and then release ***p***_*i*_.

**Output:** Each participant *P*_*i*_ receives the partially decrypted result {***p***_*j*_}_*j*≠*i*_ from others, and computes the final decryption result *μ*←MFHE.FinDec(***p***_1_,⋯***p***_*t*_).

### 6.1. Security of the protocol

Next, we will prove that the SMC protocol constructed herein is secure in the semi-malicious environment; that is to say, the protocol is secure when facing a semi-malicious adversary, who is weaker than the malicious adversary but more robust than the semi-honest adversary.

a) First, a PPT simulator *S* is designed for a semi-malicious adversary who has captured *N*−1 users. The semi-malicious adversary in the static state is marked as *A*. Assuming that *P*_*h*_ is the only surviving honest participant. On behalf of *P*_*h*_, Simulator *S* follows the steps below.

In the second round, Simulator *S* replaces the real input of the honest participant *P*_*h*_ with 0 for encryption. Subsequently, Simulator *S* obtains the inputs and secret keys of *N*−1 captured participants from "the proof tape." *S* sends these inputs into an ideal machine to get the output *y* and the ciphertext ***C*** after the homomorphic computation. Then, *S* computes and simulates the partially decrypted ***ρ***_*h*_←*S*(***y***,***C***,*h*,{*sk*_*i*_}_*i*∈[*N*]\{*h*}_) for *P*_*h*_ and discloses the partially decrypted result simulated in the third round to replace the real decryption.

By using a series of hybrid attacking games *REAL*_*π*,*A*,*Z*_, *HYB*_*π*,*A*,*Z*_, and *IDEAL*_*F*,*S*,*Z*_, it is proven that the real and simulation results are indistinguishable. The definitions and proving means of these games resemble those in Literature [6], so it is concluded that $REAL_{\pi ,A,Z}\overset{\mathrm{stat}}{\approx }HYB_{\pi ,A,Z}$ and $HYB_{\pi ,A,Z}\overset{\mathrm{comp}}{\approx }IDEAL_{F,S,Z}$. More proving details can be found in Literature [6]. It is finally proven that the real and simulation computations are indistinguishable, namely, $IDEAL_{F,S,Z}\overset{\mathrm{comp}}{\approx }REAL_{\pi ,A,Z}$.

b) Assuming that the adversary has corrupted multiple honest participants, just like Literature [6], a pseudorandom function can be adopted to prove that the protocol proposed herein is secure.

Therefore, the protocol is secure in a semi-malicious environment. The proving process ends.

## 7. Comparisons and analyses of protocol performance

Compared to the KLP18 scheme [23] that supports single-bit encryption only, the proposed protocol can support multi-bit encryption. Assuming that the information to be encrypted is in B-bit, the KLP18 scheme needs to be encrypted for B times, but only one encryption is required by adopting our protocol.

In comparison with Literature [29], the scheme in this study needs to be improved from two aspects:

a) Ciphertext size: Both the basic MGSW scheme proposed herein and the scheme offered in Literature [29] support multi-bit encryption. However, the former changes how the GSW scheme is implemented, requiring smaller ciphertext size and achieving $\frac{{\left(n+1\right)}^{2}{\left\lceil \log q\right\rceil }^{2}}{\left\lceil \log p\right\rceil }$; the latter demands a ciphertext size of (*n*+1)^2^⌈log*q*⌉^2^. For B-bit encryption and decryption, the ciphertext sizes in Literature [10] and this study are ${\left(n+B\right)}^{2}{\left\lceil \log q\right\rceil }^{2}$ and $\frac{{\left(n+B\right)}^{2}{\left\lceil \log q\right\rceil }^{2}}{\left\lceil \log p\right\rceil }$, respectively.

b) Storage overhead: Although the GSW scheme is adopted as the basic scheme for both this study and Literature [29], the former’s ciphertext size is much smaller than the latter, suggesting that our protocol dramatically reduces the ciphertext size. In this way, the protocol proposed in this study occupies a smaller memory space but offers higher overall efficiency than in Literature [25].

The comparisons of protocol performance are detailed in Table 1, where "basic" refers to the basic scheme adopted in the protocol; "multi-bit" suggests whether the scheme supports multi-bit encryption; “Storage” is the Storage overhead; n is the lattice dimension; q is the modulo; p means the power of 2; B is the bit quantity inputted.

**Table 1:** Performance comparison of schemes without CRS.

Scheme	Basic	Multi-bit	Ciphertext Size	Storage
Kim et al. [23]	GSW13	No	(*n*+1)^2^⌈log*q*⌉^2^	High
Li et al. [29]	GSW13	Yes	(*n*+*B*)^2^⌈log*q*⌉^2^	High
Ours	GSW13	Yes	$\frac{{\left(n+B\right)}^{2}{\left\lceil \log q\right\rceil }^{2}}{\left\lceil \log p\right\rceil }$	Low

## 8. Conclusion

In this study, the key generation algorithm in the FHE scheme offered by Chen Li et al. was modified to construct the MGSW scheme so that the participants do not need to rely on the CRS matrix to generate their keys. Further, the MGSW scheme and the LinkAlgo algorithm were adopted for achieving the MFHE scheme. Finally, a three-round interactive SMC protocol without CRS that supports multi-bit encryption was designed using the MFHE scheme. Its security was based on the DLWE assumption, and it was proven secure in the semi-malicious model. The protocol proposed herein outperforms the existing ones in terms of the support for multi-bit encryption, ciphertext size and storage overhead, and functions more efficiently as a whole.

Yet, all existing FHE-based SMC protocols without CRS were proven to be secure in the semi-malicious environment only, but cannot resist the attack of a malicious adversary [34]. How to construct an SMC protocol without CRS in a malicious environment remains to be solved, indicating our future research direction.
